# Clinical and pharmacokinetics overview of intranasal administration of fentanyl^☆^

**DOI:** 10.1016/j.heliyon.2023.e23083

**Published:** 2023-12-01

**Authors:** Samaneh Nakhaee, Farhad Saeedi, Omid Mehrpour

**Affiliations:** a Medical Toxicology and Drug Abuse Research Center (MTDRC), Birjand University of Medical Sciences (BUMS), Birjand, Iran; b Student Research Committee, Birjand University of Medical Sciences, Birjand, Iran; c Michigan Poison & Drug Information Center, Wayne State University School of Medicine, Detroit, MI, USA; d AI and Health LLC, Tucson, AZ, USA

**Keywords:** Intranasal, Fentanyl, Intranasal opioid, Nasal rout

## Abstract

Due to the presence of large surfaces and high blood supply, drug delivery through the nasal route of administration is the appropriate route to administrate drugs with rapid onsets of action. Bypassing first-pass metabolism can increase drug bioavailability. The physicochemical properties of fentanyl led to a need to develop formulations for delivery by multiple routes. Several approved inter-nasal fentanyl products in Europe and the USA have been used in prehospital and emergency departments to treat chronic cancer pain and used to treat severe acute abdominal and flank pain. Analgesia durations and onsets were not significantly different between intranasal and intravenous fentanyl in patients with cancer breakthrough pain and were well-tolerated in the long term. Intranasal Fentanyl (INF) at a 50 μg/ml concentration decreased renal colic pain to the lowest level in 30 min. Possible adverse effects specific to INF are epistaxis, nasal wall ulcer, rhinorrhea, throat irritation, dysgeusia, nausea, and vomiting. However, there is limited available literature about the serious adverse effects of INF in adults and children. Intranasal Fentanyl Spray (INFS) results in significantly higher plasma concentrations and has a lower T_max_ than oral transmucosal formulation, and the bioavailability of fentanyl in intranasal formulations is very high (89 %), particularly in pectin-containing formulations such as PecFent and Lazanda.

## 1 Background

Fentanyl is an opioid agonist with 50–100 times higher potency than morphine in analgesic effect that is frequently used to treat acute and chronic pain [1,2]. The physicochemical properties of fentanyl lend to a desire to develop different formulations for delivery by different routes [3]. Owing to a large surface and high blood supply, nasal drug delivery is the appropriate route to administrate the drug, which has a rapid onset of action. Not relying on first-pass metabolism (i.e., not being a prodrug) increases bioavailability. Also, ease of administration and non-invasiveness increase patient acceptability [1,4]. INF has garnered recognition for its swift onset of action and efficacy, particularly in managing breakthrough cancer pain. Its intranasal route offers a non-invasive mode of administration, presenting an advantageous option for patients with dysphagia or in situations where intravenous (IV) access is challenging. Furthermore, it is noteworthy that patients presenting with severe acute abdominal and flank pains often necessitate referral to the emergency department [5,6].

INF is a suitable and effective analgesic method in children. The non-invasive method of drug delivery and the absence of bad taste in nasal formulations make this form better accepted than oral formulations in children. According to studies performed on children aged 1 to 3 with moderate to severe pain, no adverse drug reactions or side effects have been identified and are well tolerated by children [7].

Intranasal Fentanyl Spray (INFS) is available in doses of 100, 200, 400, or 800 μgrams, using industrial sprays at 100 or 400 μgrams per spray and containing eight doses. Initial doses should be 100 μgrams in all patients regardless of prior opioid use [7]. Several approved INF products in Europe [1] and the USA [8] have been utilized in emergency departments and prehospital settings.

This study compares INF's efficacy, disadvantages, and pharmacokinetics with other formulations. In this narrative review, we conducted a comprehensive literature search using the PubMed, Web of Science, and Scopus databases, using the keywords ‘intranasal fentanyl’, 'pharmacokinetics', ‘clinical trials', and ‘bioavailability’. We included studies that investigated the pharmacokinetics, clinical efficacy, and safety of intranasal fentanyl. Studies were excluded if they were not in English, did not involve human subjects, or did not provide specific data on intranasal fentanyl. Books, letters to the editor, and nonclinical studies were also excluded.

**Fig. 1:**
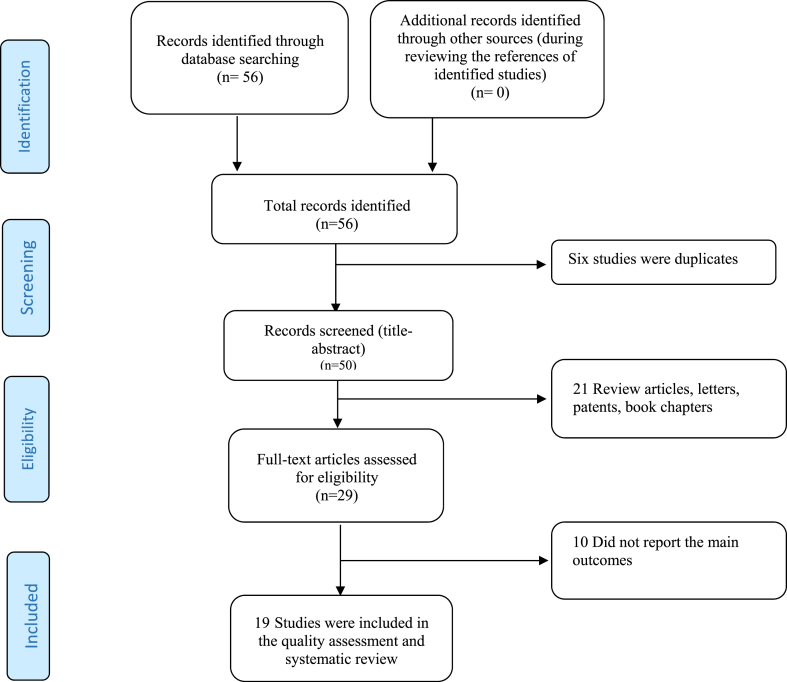
PRISMA Flowchart of the literature search and strategy for selecting the relevant documents.

The data from these studies were extracted. An electronic data abstraction form was used for recording study characteristics, including first author name, year of publication, type of study, sample size, mean age of participants, sex, doses of INF used, the incidence of adverse events for each trial, and the main results of the study. In total, we reviewed 19 clinical trials involving a total of 3469 patients. Fig. 1 shows the Flowchart of the literature search of our study. The key findings from these trials are summarized in Table 2. A standardized checklist was employed to assess the quality of the studies incorporated in the review. Two independent reviewers critically appraised each study utilizing the Joanna Briggs Institute (JBI) Checklist for Systematic Reviews (CSR). The CSR comprises questions that are answered with “yes”, “no”, or “unclear”. A response of “yes” is awarded 1 point, while “no” or “unclear” responses receive 0 points [9]. Each identified study meeting our study criteria was excluded based on the results of the quality assessment stage.

**Table 1:** Pharmacokinetics properties of approved intranasal fentanyl products.

Product	Instanyl®			PecFent®/Lazanda®	
**Dose**	75, 100, 150, or 200 μg [9,25]	200 μg [53]	75, 100, 150, 200 μg [37]	100, 200, 400, and 800 μg [24]	100 μg [54]
**T**_**max**_	12–15 min	shorter T_max_ compared to OTFC	10.8, 11.4, 15.7, and 13.8 min for 75–200 μg respectively	20,15, 21, 20 min for 100–800 μg, respectively	19.8 min
**C**_**max**_	0.35–1.2 ng/ml for fentanyl 50–200 μg	815 pg/ml	0.7, 1, 1.4, and 1.7 ng/ml for 75–200 μg respectively	352, 781, 1552, 2844 pg/ml for 100–800 μg, respectively	337 pg/ml
**T**_**1/2**_	3–4 h	–	89, 178.8, 176.7, and 116 min for 75–200 μg respectively	15–24.9 h	9.7 h
**Relative Bioavailability**	^a^89 %	^b^115 % (2.45-fold higher for INFS compared to OFTC)	^a^89 %	^b^103 %, 119 %, 163 %, and 161 % for 100–800 μg, respectively	^b^133 %

a: Relative bioavailability to intravenous fentanyl.

b: Relative bioavailability to oral transmucosal fentanyl citrate 200 μg.

**Table 2:** Study characteristics of included studies for the clinical efficacy of INF.

Author (Year)	Country	Study type	Age	Gender	INF dose	Population	Groups	Sample size	Outcome	Side effects
Nazemian (2019) [5]	Iran	RCT	–	56.4 % male	2 μg/kg	Patients with severe renal colic	IV fentanyl	220	Pain decreased compared to IVF	Nausea, Dizziness, Pruritus, Respiratory depression
Banala (2020) [29]	USA	RCT	52.9	–	100 mcg	Severe pain of cancer	IV hydromorphone	82 INF = 42 IVH = 40	shorter time to administration of INF	–
Mercadante (2009) [32]	Italy	RCT	62.0	56.8 % male	50, 100 or 200 μg	Cancer-related breakthrough pain	oral transmucosal fentanyl citrate	139 INF = 71 OTMFC = 68	Pain decreased compared to oral transmucosal fentanyl citrate	Nausea, Constipation, Vomiting, Malignant neoplasm progression, Diarrhea, Dizziness, Asthenia, Urinary tract infection, Pyrexia, Dyspnoea, Somnolence, Dysgeusia
Kress (2009) [33]	Austria	RCT	60.6	50.5 % male	50, 100, or 200 μg	Cancer-related breakthrough pain	INF	INF = 111 Placebo = 110	Pain decreased from baseline	Malignant neoplasm progression, Vertigo, Nausea, Constipation, Vomiting, Anemia, Asthenia, Peripheral edema, Decubitus ulcer
Taylor (2014) [34]	USA	RCT	53.5	52.0 % male	100–800 μg	Cancer-related breakthrough pain	–	163	INF was well tolerated	Insomnia, Nausea, Vomiting, Peripheral edema, Anemia, Constipation, Diarrhea, Asthenia, Pain, Dyspnea, Pyrexia, Cancer pain, Urinary tract infection, Anxiety, Chest pain, Anorexia, Depression, Pneumonia, Weight decrease, Back pain, Cough, Dizziness, Breast cancer
Mercadante (2015) [36]	Italy	Prospective cohort study	64.8	65 % male	87–119 μg	Cancer-related breakthrough pain	–	75	INF was well tolerated	Headache, Drowsiness, Constipation, Dizziness
Christrup (2008) [37]	Denmark	RCT	24.1	46 % male	75, 100, 150, or 200 μg	Third-molar extraction.	IV fentanyl	24 Each participant received INF and IVF	There were no differences between INF and IVF regarding onsets and durations of analgesia	Vertigo, Dizziness, Nausea, vomiting, concentration impaired, Emotional lability, Appetite decreased, Respiratory depression, Itching, Vasospasm
Karlsen (2014) [38]	Denmark	Prospective observational	58	46 % male	50–300 μg	Orthopedic conditions, acute coronary syndrome refractory to nitroglycerin spray, abdominal pain		903	Pain decreased	4 %) experienced mild adverse effects, including vomiting, mild hypotension, nausea, abdominal pain, vertigo, or decrease of Glasgow Coma Scale score to 14, and rash
Belkouch (2015) [39]	Morocco	Prospective study	51.3	52.2 % male	1.5 mg/kg and 50 mg/ml concentration	Renal colic	–	23	Pain decreased from baseline	–
Crellin (2010) [40]	Australia	Prospective, observational study	8.9 years	90 % male	1.5 μg/kg and 50 μg/ml	Upper limb injuries		59	Pain decreased compared to IVF	No adverse effect
Valtola (2021) [49]	Finland	RCT	63 (100 μg) 65 (200 μg)	81 % male	100 μg and 200 μg	Cardiac bypass surgery patients	–	16	Pain decreased from baseline	Nausea, vomiting, confusion
Thronæs (2015) [50]	Norway	RCT	61.0	32.6 % male	50, 100, 200 or 400 μg	Cancer-related breakthrough pain	Placebo	46	Pain decreased	dizziness, nausea, fatigue, vomiting, abdominal pain, Hot flush, Hypoesthesia, Headache, Somnolence, Hyperhidrosis, Nasal discomfort, Nasal edema, Vertigo, Malignant neoplasm progression
Kaasa (2010) [51]	Norway	RCT	–	–	50/100, 100/50, 50/200, 200/50, 100/200, and 200/100 μg	Cancer-related breakthrough pain	–	19	Short T (max) for 50, 100, and 200 μg INF	31.6 % of patients experienced adverse outcomes, the majority being mild in severity
Tanguay (2020) [42]	Canada	Retrospective analysis	59.0 ± 19.9	Male: 48.4 %	patients <70 years old: 1.5 μg with a maximum dose of 100 mcg Patients >70 years old: 50 mcg	Patients with acute severe pain	Subcutaneous fentanyl	1440 INF = 762 SCF = 678	IN and SC fentanyl are safe and effective	Ramsay level >3 Respiratory rate <12 breaths/min Pulse <50 P/min SpO2 < 90 %
Paech (2003) [55]	Australia	pilot study	42	–	50 μg	gynecological patients	IV fentanyl	24 Each participant received INF and IVF	rapid onset (within 5 min) of analgesia	mild nasal stinging
Fisher (2010) [44]	UK	RCT	–	–	100 μg	opioid-naïve, healthy adult	oral transmucosal fentanyl citrate	18	Lower peak plasma time compared to oral transmucosal	Nasal reactogenicity symptom incidence was lowest for the FPNS
Fisher (2010) [24]	UK	RCT	–	–	100, 200, 400, and 800 μg	opioid-naïve	oral transmucosal fentanyl	16	shorter tmax, higher Cmax, and greater bioavailability compared to oral transmucosal fentanyl	
Borland (2005) [45]	Australia	RCT	4.5	71 % male	1.4 mg/kg	Children with burn injury	oral morphine	24 INF = 10 OM = 14	INF was equivalent to oral morphine	Itching, abdominal cramps, headache, drowsiness
Borland (2007) [46]	Australia	RCT	10.9	–	150 μg/ml	Children with acute long-bone fractures	IV morphine	67 INF = 33 IVM = 34	INF was effective compared to IV morphine	Vomiting

RCT: randomized clinical trial; INF: intranasal fentanyl; IVF: intravenous fentanyl, IVH=IV hydromorphone, OTMFC = oral transmucosal fentanyl citrate, SCF= Subcutaneous fentanyl.

### 1.1 Characteristics of fentanyl formulations for nasal delivery

Fentanyl, an opioid with high lipophilicity, can easily pass through biological membranes, including transmucosal membranes [10]. Various transmucosal formulations have been authorized for pain management. These formulations utilize absorption through the buccal, sublingual, or nasal mucosa to ensure quick entry into the bloodstream and bypass metabolism by the liver in the first pass [10]. In Europe, two nasal fentanyl products (Instanyl® and PecFent®) are approved, while in the USA, there is one (Lazanda®). Both are formulated at suitable concentrations for intranasal use [11].

The use of intranasal (IN) delivery is increasingly being explored as an alternative method for administering therapeutics to the Central Nervous System (CNS) [12]. Essentially, the nasal cavity offers a significant surface area (approximately 150–180 cm^2^ in humans) [13], and turbinate structures enhance the interaction between inhaled air and the mucosal surface. The nose provides a direct pathway to the CNS through the olfactory route. Theoretically, this allows for direct drug delivery to the brain, enabling lower drug doses, minimizing exposure to non-targeted organs, and potentially reducing toxic side effects. The nasal epithelial membrane is thin, well-supplied with blood, facilitates good venous outflow, and has leaky intercellular junctional complexes [1,12,[14], [15], [16], [17]]. These anatomical characteristics create favorable conditions for efficient and rapid absorption of drug compounds.

Additionally, the nasal mucosa has a pH of 5.5–6.5, which optimizes the function of glycoproteins to which drugs can bind. Factors such as lipophilicity, drug ionization, and mucociliary clearance influence drug absorption through the nasal mucosa. Considering these attributes, the nasal route holds promise as an option for delivering drugs like lipophilic opioids [16]. Small lipophilic drugs are generally absorbed well from the nasal cavity [1,17], and their absorption speed and extent are often comparable to IV injection [1,14,15]. The nasal route of administration also allows drugs to directly reach the CNS site of action through the olfactory and trigeminal nerves, vasculature, cerebrospinal fluid, and the lymphatic system. These factors result in rapid analgesic effects on the CNS, increased therapeutic bioavailability in the CNS, reduced peripheral side effects, and lower dosage requirements [1,12,14]. The nose-to-brain route offers advantages by bypassing common obstacles encountered in oral CNS drug delivery, such as gastrointestinal pH and enzymes, variable absorption, hepatic drug metabolism, serum-associated degradation, kidney filtration, and the blood-brain barrier [12,17]. Furthermore, when considering patient care, intranasal administration presents numerous advantages: it is non-invasive, convenient, and easy for patients to self-administer. This method of delivery may be particularly advantageous for individuals with movement disorders, those experiencing impaired gastrointestinal function, or those suffering from dry mouth as a result of salivary gland dysfunction [1,12,14].

IN administration is a valuable option when it is challenging to achieve successful intravenous cannulation, when oral dosing is problematic due to nausea and vomiting, or when circumstances do not allow for intravenous pain management [14]. Plasma concentrations measured after intranasal administration are similar to concentrations after IV injection [1,14]. The bioavailability of INF ranges from 55 % to 77 % [18], comparable to oral transmucosal but higher than oral administration [17]. It has a rapid onset of action (7 min), with a peak effect occurring at 12–15 min and a duration of approximately 2 h [14]. These pharmacokinetic parameters make it a favorable choice for managing breakthrough cancer pain and acute pain, especially in out-of-hospital care and among the pediatric population, due to its non-invasive nature and quick onset of action [14].

The Instanyl INFS is a medication containing fentanyl citrate dissolved in a buffered solution. It is administered through a nasal spray device, either as a single dose or multiple doses. Fentanyl citrate is a combination of fentanyl and citric acid, and when the spray is applied to the nasal cavity, fentanyl is absorbed into the bloodstream through the nasal lining. The absorption is quick, with maximum arterial concentration reached within 7 min. The onset of pain relief typically occurs within 7–10 min, and the effects last for approximately 60 min when given as a single dose [10,19]. However, the amount of drug absorbed can vary since some of the sprayed volumes may drain into the throat and be swallowed, undergoing metabolism in the liver [10,14]. The pH of the formulation is important for efficient nasal absorption [17], and certain additives such as citric acid and sodium phosphate are used as buffers to maintain the pH, reducing nasal irritation and regulating the absorption rate of the active ingredient [1,12].

To improve the absorption of nasal sprays and reduce irritation, newer formulations like NasalFent, PecFent, and PecSys have incorporated additives such as pectin [14]. These additives create a thin gel layer on the nasal lining. The Fentanyl Pectin Nasal Spray (FPNS), or PecFent, utilizes the PecSys drug delivery system based on pectin [19]. It is an aqueous solution with low viscosity that contains fentanyl citrate. When sprayed into the nasal cavity, it forms a gel that allows fentanyl to be absorbed [10,19]. Compared to INFS, the drug is less likely to run into the throat [10,14]. The formulation includes pectin as a viscosity enhancer, which helps prolong its stay in the nasal cavity and prevents it from dripping down the throat, enhancing its effectiveness. Other excipients like phenylethyl alcohol and methylparaben are included to improve the formulation and enhance IN delivery [12].

### 1.2 Drug delivery to the brain after intranasal administration

In human adults, the nasal cavity holds around 15–20 ml of space, covered by a surface area of approximately 150–180 cm^2^. Out of this surface area, about 5–10 cm^2^ is olfactory epithelium, while the remaining 145–170 cm^2^ is respiratory epithelium. The olfactory nerve appears to serve as a direct pathway into the CNS, referred to as the olfactory vector hypothesis [9]. By following this pathway, even opioids with a slow blood–brain barrier crossing rate can quickly produce analgesic effects in the CNS [9]. The absorption of fentanyl through the nasal route occurs via two pathways: firstly, it can be absorbed into the bloodstream through the abundant nasal vascular beds, and secondly, it can directly enter the brain through the exposed olfactory and trigeminal nerves in the nasal cavity [20].

**Fig. 2:**
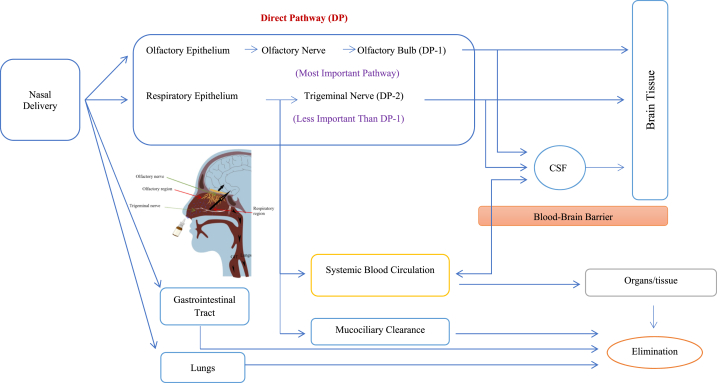
The potential routes of drug delivery to the brain after intranasal administration.

Potential routes for direct access to the CNS include the olfactory and trigeminal nerves, the blood vessels, the cerebrospinal fluid, and the lymphatic system [9]. Therapeutics mainly travel from the nasal epithelium to the CNS through the olfactory and trigeminal nerve pathways, using intracellular- and/or extracellular-dependent processes. However, depending on the specific properties of the therapeutic and its accompanying substances, one particular pathway and mechanism may dominate the transport process. Additionally, some therapeutics may be absorbed by nearby blood and lymphatic vessels and distributed throughout the body, with a small portion eventually reaching the brain through an indirect route [12] (Fig. 2).

### 1.3 Pharmacokinetics of intranasal fentanyl

When fentanyl is given through the nose, about 70 % of it is absorbed into the body, with the highest concentration reached within 5–16 min [16]. Its effect lasts for up to 65 min when administered intranasally. The absorption rate of fentanyl is affected by pH, with higher pH levels leading to increased absorption [16]. Temperature also plays a role, as warmer temperatures enhance the permeation of fentanyl through the nasal route. Compared to administering fentanyl intravenously, the intranasal method takes longer to produce an effect.

Additionally, the half-life of fentanyl is longer when given intravenously compared to intranasally [16,21]. Fentanyl mostly binds to α-1-acid glycoprotein but also attaches to albumin and lipoproteins, making up approximately 80–85 % of its protein binding. It has a relatively large volume of distribution, averaging at 4 L per kilogram of body weight, ranging from 3 to 8 L per kilogram. Fentanyl is highly lipophilic and can easily cross the blood-brain barrier once it enters the bloodstream [22]. In the hepatic system, fentanyl is metabolized by the enzyme CYP3A4, resulting in the formation of an inactive metabolite known as norfentanyl [22]. The intranasal route reduces drug metabolism and increases the duration of the drug effects [9].

Fentanyl is primarily eliminated through urine, with more than 90 % of the original drug excreted as inactive metabolites undergoing N-dealkylation and hydroxylation. Approximately 7–10 % of the drug is excreted unchanged as an active medication [22].

In developing INFS for adult patients undergoing dental surgery, the pharmacokinetics of different doses (75, 100, 150, and 200 μg) were studied using buffered aqueous solutions with a pH range of 6.3–6.4. The mean maximum concentration (C_max_) of fentanyl in the blood was 0.7, 1.0, 1.4, and 1.7 ng/ml for the respective doses. The absorption rate was rapid, with a mean time to reach maximum concentration (T_max_) ranging from approximately 11 to 16 min. However, the report did not mention the bioavailability of the drug [21]. Nave et al. showed that C_max_ and AUC_0 –∞_ of INFS for single- and multi-dose delivery systems were 948 pg/ml, 949 pg/ml, and 4439 pg × h/ml, 4489 pg × h/ml, respectively, in 48 patients receiving 200 μg/100 μg [23].

During a pharmacokinetic study of FPNS, participants were given different doses of fentanyl ranging from 100 to 800 μg. These doses were compared to a standard dose of Oral Transmucosal Fentanyl Citrate (OTFC) at 200 μg. The maximum concentration (C_max_) of fentanyl in the blood increased proportionally with the dose, with mean values of 0.35, 0.78, 1.55, and 2.84 ng/ml for the four FPNS doses—the duration required to achieve peak concentration denoted as Tmax spanned from 15 to 21 min. Concurrently, the bioavailability of FPNS, when compared to OTFC, exhibited a variation ranging from 103 % to 163 % [24].

INF exhibits rapid absorption, with a bioavailability of over 70 %. Its distribution, metabolism, and elimination are consistent with other administration routes. All formulations have almost the same C_max_ and T_max_ (12–21 min). One notable exception is PecFent®/Lazanda®, which has a reported bioavailability of over 100 %. Due to the use of pectin in PecFent/Lazanda formulations, the half-life of these products is longer (15–25 h) than Instanyl (3–4 h). Also, Instanyl's time to onset is shorter than for pectin-containing formulations [9,25,26].

Several research studies have shown that the relative bioavailability of OTFC exceeds 100 %. These bioavailability data suggest that Fentanyl Pectin Nasal Spray (FPNS) and OTFC, although containing equivalent fentanyl dosages, may not provide comparable analgesic effects [22]. This discrepancy is attributed to the unique formulation and delivery system inherent to these products. These characteristics enhance the absorption of fentanyl and ensure a more consistent delivery of the drug.

The primary purpose of the PecSys gelling technology used in FPNS is to control the absorption of fentanyl and regulate the peak concentration in the bloodstream. The gelling allows for a longer duration of fentanyl exposure while achieving an early maximum concentration (T_max_), which aligns with the typical rapid onset and short duration of breakthrough pain (BTP) episodes. By controlling the rate and extent of fentanyl absorption from the nasal cavity, the PecSys technology enables FPNS to provide analgesia tailored to BTP [27]. In vitro studies using diffusion cells have shown that FPNS effectively controls the release of fentanyl compared to a simple solution [28]. When comparing the pharmacokinetic data of FPNS to simple fentanyl solutions, it is evident that FPNS produces a lower maximum fentanyl concentration (C_max_) for an equivalent dose. The lower C_max_ of FPNS demonstrates the effectiveness of the PecSys technology in modulating fentanyl absorption in vivo [11]. The summary of pharmacokinetics properties of approved INF products is shown in Table 1. The increased bioavailability of PecFent®/Lazanda® is because fentanyl is absorbed directly into the bloodstream through the nasal mucosa and lung epithelium, bypassing the liver's first-pass metabolism. This results in higher bioavailability than other forms, such as oral tablets or transdermal patches. Incomplete absorption and pre-systemic elimination via CYP3A both reduce the bioavailability of formulations that deliver fentanyl into the gastrointestinal tract. Additives such as pectin have been included in these newer spray formulations to improve nasal penetration and reduce local irritation [15]. However, there may be concerns about potential side effects associated with rapidly reaching very high plasma concentrations with simple solutions for nasal administration in a clinical setting [28]. The PS gelling technology used in FPNS aims to match the absorption profile to the typical time course of breakthrough pain episodes by modulating fentanyl absorption, reducing peak plasma concentration, and prolonging overall exposure while allowing an early T_max_ [11]. On an equivalent dose basis, FPNS generates a lower C_max_ than simple solutions or non-gelling chitosan formulations, demonstrating the ability of PecSys to modulate absorption in vivo [11]. PecSys is a patented gelling drug delivery system designed for mucosal surfaces such as the nasal cavity, eye, and vagina. Pectin, a well-defined plant-derived polysaccharide, is used as an additive in newer spray formulations due to its excellent regulatory position and long history of pharmaceutical and food usage [28]. PS forms a gel on contact with the mucosal surface due to the interaction between pectin and calcium ions in the mucosal fluid, allowing locally acting drugs to remain at the application site for longer periods. Gels formed from pectin can modulate systemic uptake and control pharmacokinetic profiles for well-absorbed (lipophilic) drug compounds. PS solutions have low viscosity and can be given in low volumes for nasal delivery (e.g., 0.1 ml or less) [28]. Another crucial aspect of a PS formulation is its ability to consistently deliver the same dose and maintain the same spray characteristics, including droplet size, spray pattern, and plume geometry. Although calcium concentrations may affect pectin gel consistency, diffusion characteristics of gelled PS formulations are tolerant to calcium concentration-related changes in gel properties, resulting in relatively unaffected in vivo performance of PS formulations even in the event of local changes in nasal calcium concentrations [28].

## 2 Clinical efficacy

The onset of action is faster in INF than for oral formulations. Analgesic potency in IV administration is higher than in INF, but patient compliance with the IN route is higher, especially in children [29].

BTP is a transient pain common in cancer patients. The BTP prevalence among cancer patients is estimated at 19–95 %. Fentanyl is one of the drugs used to control BTP. In addition, many studies have been performed on the clinical effect of INF to control BTP [30]. Because oral transmucosal or INF has a rapid onset of action, sustained effect, and potent analgesic effect, it can be considered one of the efficient treatments for BTP [31].

Lyseng et al. conducted two double-blind, randomized trials among opioid-tolerant adults and found that FPNS at 100–800 mg significantly reduced pain intensity compared to placebo. In addition, it relieved pain significantly faster than oral immediate-release morphine. In an open-label, 16-week trial, FPNS was shown to be an effective pain reliever during long-term treatment of BTP. In addition, FPNS 100–800 mg was commonly well-tolerated with no nasal tolerability complications [27].

A randomized, crossover, open-label trial investigated the effectiveness of INF spray and OTFC in treating BTP in cancer patients. In this study, a total of 196 patients were enrolled, out of which 139 were randomly assigned to receive either INF spray followed by OTFC or vice versa. Effective doses of INF spray (at 200, 100, or 50 mcg) or OTFC (at 1600, 1200, 800, 600, 400, or 200 mcg) were administered to the patients to treat six episodes of BTP. The primary outcome measured was the time it took for patients to experience “meaningful” pain relief, while the secondary outcome was the difference in pain intensity. The trial showed that INF spray provided faster and more significant pain relief than oral transmucosal fentanyl citrate, and more patients preferred INF spray due to its ability to provide “meaningful” pain relief [32].

Kress et al. conducted a 10-month follow-up trial to investigate INF spray's efficacy and long-term tolerability (at 50, 100, or 200 mcg) in 120 cancer patients already tolerating opioids to treat background pain. The study aimed to evaluate the effectiveness of INF spray in treating BTp in opioid-tolerant cancer patients. The study endpoint was based on differences in pain intensity measured 10 min post-administration using a numeric rating scale. The results indicated that INF spray, administered at all three doses, effectively treated BTP in these patients and had a faster onset of action than a placebo. Moreover, all doses were clinically effective and well tolerated by the patients [33].

Taylor et al. conducted a similar study to the previous one, which aimed to explore the impact of long-term use of FPNS in treating BTP in cancer patients who were using regular opioid therapy. The inclusion criteria were adult patients who experienced one to four episodes of BTP per day and were taking at least 60 mg/day of oral morphine or equivalent. A total of 171 patients continued into the extension study. Of these, 163 used FPNS for a mean duration of 325 days. The study found that FPNS was potent and well-tolerated for long-term use in treating BTP in cancer patients [34].

Radbruch et al. conducted a study to assess the long-term tolerability, acceptability, and consistency of the effects of FPNS in patients with cancer who had BTP. The study included patients with cancer who experienced one to four episodes of BTP per day and were taking ≥60 mg/day of morphine (or equivalent). The safety and tolerability of FPNS were evaluated using adverse drug reactions, adverse events, withdrawal due to adverse events, and nasal assessments. The study included 403 patients (42,227 episodes) in the safety and intent-to-treat analysis. The results showed that almost 25 % of the patients experienced mild to moderate adverse events, but FPNS was generally well-tolerated for treating BTP. Furthermore, the doses of FPNS remained stable throughout the 4-month study period [35].

Mercadante et al. conducted a six-month observational, prospective cohort study to follow patients with advanced cancer who experienced BTP and received INF spray at effective doses. The study's primary aim was to determine whether any serious adverse effects were associated with the long-term use of INF spray. The study also aimed to evaluate the efficacy of INF spray in treating BTP, the rate and reasons for INF spray discontinuation, and sleep quality. The study included 75 patients, with a mean INF spray dose of 87–119 μg and a mean opioid dose (oral morphine equivalents) of 111–180 mg/day. The data showed that the long-term use of INF spray was safe and effective in advanced cancer patients; no serious adverse effects were observed, and the patients reported good sleep quality. Furthermore, INF spray was well-tolerated, with no treatment discontinuations due to INFS-related adverse effects [36].

A clinical trial study from July to October 2020 compared the analgesic efficacy of INF (at 100 mcg) versus IV hydromorphone (at 1.5 mg) in cancer patients with severe pain. The study showed no significant differences between the two treatments regarding their analgesic effects [29]. In a double-blind, randomized study, patients were administered one of four doses (75, 100, 150, or 200 mcg) of fentanyl through intranasal and IV routes to evaluate the efficacy and tolerability of the drug. The study found no significant difference between the duration and onset of the effects of single doses of intranasal and IV fentanyl. Additionally, the administration of the drug was generally well-tolerated through both routes [37]. In a prospective observational study, 1 to 3 doses of either 50 or 100 mg INF were administered to children older than eight years and adults with severe pain, and pain scores of 0–10 were used before and after treatment [38].

The study results of Belkouch et al. showed INF at the concentration of 50 μg/ml and dosage of 1.5 μg/kg in adult patients with renal colic decreased pain score to ½ in 5 min and reached the lowest level of pain in 30 min [39]. Also, the Crellin et al. study's results confirm that INF administration at the same dose in 59 children with upper limb injuries can reach a pain score to the lowest level in 30 min [40]. Also, in one cohort study, a neonatal intensive care unit-admitted neonate was followed for two years. Twenty-three patients received 1.3mcg/kg fentanyl through intranasal for pain management. The pain was assessed by using a premature infant pain profile (PIPP) (range 0–21, <6 = no pain). The results indicated that the pain score in the INF group during and after was low, and the mean score reached 4.3 and 3.6, respectively [41].

Tanguay et al. conducted a retrospective study on patients with acute severe pain using INF and subcutaneous fentanyl. The dosage of INF was 50 mcg for patients younger than 70 and 1.5 μg/kg with a maximum dose of 100 mcg for patients older than 70. They found both INF and subcutaneous fentanyl safe and effective. However, some adverse effects were Ramsay level >3, respiratory rate <12, breath/min pulse <50 P/min, and SpO2 < 90 % [42]. To put the onset of action in context, Peach et al. found that INF was faster than IV fentanyl with a side effect of mild nasal stinging [17].

In 2 randomized controlled trials, Fisher et al. concluded that INF has lower peak plasma, shorter T_max_, higher C_max_, and greater bioavailability than oral transmucosal fentanyl [43,44].

Borland et al. conducted two randomized controlled trials among children with burn and bone injuries to compare the efficacy of INF with oral and IV morphine. They found that INF was effective compared to IV morphine; however, INF was equivalent to oral morphine [45,46].

## 3 The adverse effect of INF

Possible adverse effects of INF are epistaxis, nasal wall ulcer, rhinorrhea, throat irritation, dysgeusia, nausea, and vomiting. However, there is no evidence of serious adverse effects of INF in clinical studies [4]. In a cohort study by McNair et al., physiological parameters were assessed in infants. Heart rate decreased, and FiO2 increased significantly 6 h after INF administration. Additionally, symptoms of respiratory depression were observed in six infants; however, ventilation was not required following the administration of the initial dose. There were no notable changes in other parameters before and after the use of Intranasal Fentanyl (INF). A repeat dose of 1.1 mcg/kg was necessary for five infants, while an increased oxygen concentration was required for one infant [41].

Another cohort study conducted to evaluate INF use in infants with a mean dose of 1.46 mcg/kg as sedation and analgesia for the invasive procedure showed no respiratory adverse event directly related to INF, and no patient required naloxone as an antidote [8]. In Karlsen's study, no serious adverse effects were observed with the treatment. Only 4 % of the patients experienced mild complications, such as vertigo, mild hypotension, nausea, vomiting, rash, abdominal pain, or a decreased Glasgow Coma Scale score of 14. The study also reported a median reduction in pain score of 3 (interquartile range of 2–5) after the administration of fentanyl [38]. Harlos et al. conducted a study involving 11 patients who were administered INF. The study found no evidence of drug-related apnea or chest wall rigidity, and INF was well-tolerated by the patients overall [47].

Portenoy et al. carried out a multicenter, open-label study that included cancer patients dealing with pain. These patients were undergoing treatment with oral morphine, or an equivalent, at a dosage of 60 mg/day or more, and they experienced 1–4 episodes of breakthrough pain (BTP) daily. The study found that the adverse effects associated with FPNS use for BTP were similar to opioids, and no nasal toxicity was observed [48]. Valtola et al. used INF with a dose of 100 μg or 200 μg to reduce the PTP in patients with cardiac surgery and found that INF had high bioavailability with a fast absorption time [49].

Thronæs et al. employed Intranasal Fentanyl Spray (INFS) at a dose of 400 μg to evaluate its efficacy in managing breakthrough pain (BTP) induced by cancer. The INFS demonstrated effectiveness, culminating in a reduction in pain intensity quantified at 2.5, with a 95 % confidence interval (CI) ranging from 1.42 to 3.49 [50]. Kaasa et al. used six different dosages of INFS (50/100 μg, 100/50 μg, 50/200 μg, 200/50 μg, 100/200 μg, and 200/100 μg) to treat BTP in patients with cancer and found that 50 μg, 100 μg, and 200 μg INFS had a low T_max_ with a high tolerability effects [51].

Moreover, adding INFS and FPNS to the base treatment of BTP with opioids could decrease the pain intensity by more than 33 % [52].

## 4 Conclusion

Oral transmucosal and intranasal are the most suitable drug delivery methods for rapid analgesic effects. Administration of opioids using the nasal route has a faster effect on the CNS. INF is an analgesic therapy for acute and chronic pain and can cause a rapid-onset therapeutic effect.

Nasal administration of fentanyl circumvents first-pass metabolism. Intranasal administration reduces drug metabolism and increases the duration of the drug. Clinical studies show that INF can have an analgesic effect in about 5 min and minimize pain in 30 min. Common side effects of fentanyl include epistaxis, nasal wall ulcer, rhinorrhea, throat irritation, dysgeusia, nausea, and vomiting, but studies have shown no serious side effects in patients with acute pain, children, and infants after receiving fentanyl intranasally. Approved INF products in Europe and the United States are available. They quickly achieve effective plasma concentrations, and the active duration is more than 1 h. Also, the bioavailability of fentanyl in intranasal formulations is higher in pectin-containing formulations.

## Funding

This research received no specific grant from funding agencies in the public, commercial, or not-for-profit sectors.

## Data availability statement

No data was used for the research described in the article.

## Ethics approval

Review and/or approval by an ethics committee was not needed for this study.

## CRediT authorship contribution statement

**Samaneh Nakhaee:** Writing – original draft. **Farhad Saeedi:** Writing – original draft. **Omid Mehrpour:** Conceptualization.

## Declaration of competing interest

The authors declare that they have no known competing financial interests or personal relationships that could have appeared to influence the work reported in this paper.
